# Optimization of Sodium Alginate Concentration and Evaluation of Individual Versus Group In Vitro Culture of Porcine Preantral Follicles in a Serum-Free Medium

**DOI:** 10.3390/ani16030376

**Published:** 2026-01-25

**Authors:** Alfredo González-Gil, Belén Sánchez-Maldonado, Carlos García-Artiga, Pedro José Aranda, Rosa Ana Picazo

**Affiliations:** 1 Department of Physiology, School of Veterinary Medicine, Complutense University of Madrid, 28040 Madrid, Spainrapicazo@ucm.es (R.A.P.); 2 Department of Animal Medicine and Surgery, School of Veterinary Medicine, Complutense University of Madrid, 28040 Madrid, Spain; belenmal@vet.ucm.es (B.S.-M.); pedaran@vet.ucm.es (P.J.A.)

**Keywords:** alginate, porcine, preantral follicle, steroid hormones

## Abstract

The increasing biomedical and conservation interest in porcine species has driven the development of advanced in vitro follicle culture systems designed to preserve genetic diversity and accurately model key stages of folliculogenesis. This study assessed a three-dimensional (3D) alginate-based system for the in vitro culture of porcine preantral follicles, aiming to overcome the structural limitations of conventional two-dimensional (2D) methods. A total of six experimental groups were established, consisting of group-cultured (four follicles/well) or individually cultured (one follicle/well) follicles maintained either without alginate (0%) or encapsulated in 0.5% or 1% alginate for 14 days in media supplemented with FSH, EGF, and IGF-I, with LH added from day 9. Follicular development was assessed by morphometric evaluation, image-based and histological analyses, and quantification of steroid hormones in media collected every 48 h. Group-cultured follicles encapsulated in 0.5% alginate most effectively maintained their 3D architecture, reached the largest diameters, and progressed more uniformly compared with other groups. In contrast, follicles cultured without alginate rapidly lost structural integrity, showed granulosa cell migration, and decreased in size, whereas those encapsulated in 1% alginate exhibited restricted growth. Estradiol and testosterone concentrations increased over time in the 0.5% alginate group, were lowest without alginate, and intermediate in 1% alginate. Individually cultured follicles exhibited reduced growth and lower total hormone production compared with group-cultured follicles; however, when normalized per-follicle, steroid secretion, particularly in the 0.5% alginate group, was enhanced, indicating increased steroidogenic efficiency on a per-follicle basis. These findings indicate that 0.5% alginate provides an optimal balance between structural support and physiological steroidogenesis during preantral follicle culture. This 3D system improves the biological relevance of porcine follicle culture and may support future applications in reproductive biology, conservation, and genetic resource preservation.

## Simple Summary

In vitro culture of porcine ovarian follicles provides an experimental model to investigate the regulation of folliculogenesis and represents a valuable tool for female gamete preservation, which is essential for maintaining swine genetic variability. Traditional two-dimensional culture systems do not offer adequate support, altering the normal structure and physiology of follicles growing in vitro. This study tested a three-dimensional alginate gel designed to form a soft scaffold around follicles. These were cultured for 14 days directly on the growth surface or in two different alginate concentrations and were monitored for their size, structure, and hormone production. Those cultured in a medium-strength alginate gel (0.5%) maintained their normal structure, achieved greater development, and produced higher hormone concentrations than follicles grown without gel (0%) or in a stiffer matrix (1%). These results indicate that a 0.5% alginate concentration offers a supportive environment that may enhance porcine follicle development in vitro and, therefore, have a positive impact on genetic conservation strategies.

## 1. Introduction

In recent years, the porcine species has gained increasing relevance in both biomedical and agricultural contexts. Due to their anatomical, physiological, and genomic similarities to humans, pigs represent valuable animal models for translational research, particularly in the field of xenotransplantation [1].

Beyond their biomedical and economic significance, the conservation of genetic diversity has become a global priority, as emphasized by the Food and Agriculture Organization (FAO) [2]. Autochthonous breeds, such as the Iberian pig, are considered critical genetic resources due to their adaptability, productivity, and biotechnological potential [3]. In this context, long-term preservation strategies, including embryo cryopreservation, have emerged as essential tools to preserve biodiversity and facilitate the international dissemination of valuable genotypes [4]. The high follicular density within the ovarian cortex also provides alternative approaches for gamete preservation, which is gaining attention as a complementary strategy in reproductive biotechnology. In particular, in vitro follicle culture serves as a robust experimental model for studying key developmental processes during folliculogenesis and offers a promising platform for the establishment of advanced fertility-preservation techniques across mammalian species [5,6].

There is a growing interest in developing in vitro systems that accurately mimic the natural microenvironment of ovarian follicles [7,8]. Although initial attempts have been made to culture isolated primordial follicles, these efforts are often limited by rapid follicle deterioration and limited growth [9]. Consequently, most in vitro folliculogenesis protocols begin at the preantral stage, which exhibits greater structural integrity and developmental competence. The in vitro culture of preantral follicles represents a promising approach for restoring fertility in women with premature ovarian failure from oncological therapies and serves as a valuable reservoir of genetic material for the recovery of endangered animal populations [10].

Two main challenges must be addressed to optimize preantral follicle culture. First, to create a more defined and controlled environment, reduce experimental variability, and minimize potential contamination risks, serum-free media are used, which must provide essential components for follicle and oocyte development, including hormones and growth factors. Gonadotropins, particularly follicle-stimulating hormone (FSH) and luteinizing hormone (LH), are critical regulators of follicular growth, estrogen synthesis, granulosa cell proliferation, and theca cell androgen production [11,12,13,14,15]. During late preantral development, follicles undergo transition from FSH-responsive to gonadotropin-dependent status, highlighting the need for LH supplementation at later stages [16]. Intraovarian factors such as EGF and IGF-I further support folliculogenesis, steroidogenesis, and oocyte maturation [14,17,18,19,20,21,22].

Second, conventional two-dimensional (2D) culture often disrupts follicular architecture, as granulosa cells attach to the substrate, causing follicle flattening and impairing cell–cell communication [7,23,24,25]. In contrast, three-dimensional (3D) culture systems have emerged as more physiologically relevant models by closely replicating the tissue microenvironment [26] through encapsulation in hydrogels such as collagen, hyaluronic acid, polyethylene glycol, or alginate [25,27]. Among these materials, alginate hydrogels are particularly effective in preserving spherical architecture and intercellular interactions due to their biocompatibility and calcium-mediated gelation [23,25,28,29]. Importantly, matrix stiffness critically influences follicle growth and steroidogenesis: softer alginate enhances expansion and hormone production, while stiffer gels may restrict nutrient diffusion [24,29,30]. Accordingly, reported optimal alginate concentrations vary among species, ranging from 0.25 to 1.5% for preantral follicles [25,29,31,32,33,34].

Recent progress in porcine follicle culture has underscored the potential of alternative 3D matrices. Specifically, materials such as agarose or alginate–fibrin composites have been shown to preserve follicular integrity, support granulosa–oocyte communication, and improve oocyte developmental competence [35,36,37]. However, direct comparisons between conventional 2D culture and alginate-based 3D systems across different alginate concentrations remain lacking, and identifying the optimal alginate concentration remains crucial for establishing in vitro culture systems capable of supporting complete folliculogenesis.

The ability of follicles to synthesize and secrete steroid hormones is widely recognized as a reliable functional biomarker of follicular viability and oocyte development potential [38] and serves as a valuable parameter for monitoring in vitro folliculogenesis. Consequently, the evaluation of steroid hormone production represents a key indicator of follicular functionality and remains of considerable interest for elucidating the physiological mechanisms underlying follicle development.

In addition to the 3D microenvironment, follicular development is substantially influenced by culture, as individual follicles versus in groups. While individual follicle culture allows precise monitoring of growth dynamics, hormone production, and structural integrity, group-cultured follicle systems offer notable advantages, such as preservation of follicle integrity, increased growth and survival, and enhanced paracrine-mediated interfollicular communication [39,40]. Therefore, understanding the combined effects of alginate concentration and the choice between individual and grouped culture is essential for optimizing in vitro systems for porcine preantral follicle development.

We hypothesize that group-cultured porcine preantral follicles in a serum-free medium supplemented with gonadotropins (FSH and LH) at defined time points, along with growth factors such as EGF and IGF-I, would provide the optimal in vitro conditions to assess the impact of 2D versus 3D culture systems. Moreover, this approach would enable the determination of the optimal alginate concentration required to support preantral follicular development within a 3D matrix.

The aim of this study was to establish an alginate-based 3D culture system that supports porcine preantral follicle growth while maintaining structural and functional integrity. For this purpose, different alginate concentrations and individual versus group-cultured follicle conditions were assessed to determine how the concentration of alginate used as a 3D scaffold and inter-follicular interactions influence follicle survival, morphology, and steroidogenic function.

## 2. Materials and Methods

### 2.1. Animals and Ovary Collection

Ovaries were obtained from prepubertal Large White–Landrace crossbred pigs (12–15 weeks old) sacrificed at a local abattoir (Matadero Comarán S.L, Aranjuez, Madrid, Spain). Samples were transported to the laboratory within 1 h of collection in sterile saline solution (2–8 °C) supplemented with 0.306 g/L penicillin (Sigma-Aldrich, ref. P3032; Saint Louis, MO, USA) and 0.680 g/L streptomycin (Sigma-Aldrich, ref. S1277).

### 2.2. Follicular Isolation, Encapsulation, and Culture

Ovaries were washed by repeated replacement of the collection medium with fresh solution. Cortical tissue was dissected on ice in a Petri dish and sectioned with a scalpel into small fragments (approximately 0.5 × 0.3 mm and <1 mm deep) from areas devoid of antral follicles. Tissue fragments were enzymatically digested in Hank’s medium with Ca^2+^ and Mg^2+^ (Sigma-Aldrich, ref. H8264) containing 0.1% collagenase type IA (Sigma-Aldrich, ref. C2674), 1% bovine serum albumin (BSA, Sigma-Aldrich, ref. A9418), 1000 IU/10 mL deoxyribonuclease (Sigma-Aldrich, ref. D4513), and 100 µL/10 mL antibiotic/antimycotic solution (Gibco, Life Technologies, ref. 15240-062; Carlsbad, CA, USA). Digestion was performed for 30 min on a shaker at 37 °C under 5% CO_2_. After centrifugation, the enzyme solution was replaced with Hank’s solution without Ca^2+^ and Mg^2+^ (Sigma-Aldrich, ref. H6648) containing 1% BSA, 5000 IU/10 mL heparin (Chiesi, Barcelona, Spain), 1000 IU/10 mL deoxyribonuclease, and 100 µL/10 mL antibiotic/antimycotic solution. Large fragments were dissociated by gentle and repeated pipetting through Pasteur pipettes of decreasing diameters. Fragments were transferred into 4-well plates (Nunclon Delta, ref. 176740; New York, NY, USA) containing complete tempered Dulbecco’s Modified Eagle Medium F-12 (DMEM/F-12, Gibco, Thermo Fisher, Waltham, MA, USA, ref. 21041025) supplemented with 1% BSA, 0.2% Synthecol (Sigma-Aldrich, ref. S5442), 3 mM L-glutamine (Sigma-Aldrich, ref. G7513), 1% insulin–transferrin–selenium (ITS, Sigma-Aldrich, ref. I3146), and antibiotic/antimycotic.

Intact preantral follicles with eight to ten layers of granulosa cells, measuring 240–260 μm in diameter [41,42], were mechanically isolated from the surrounding stroma using a 25-gauge needle under a stereomicroscope equipped with an ocular graticule to assess follicle size. Follicular cell viability was assessed by Trypan Blue staining (Sigma-Aldrich, ref. T8154).

A total of 180 viable follicles were used in the study, which was conducted in two independent experiments. In each experiment, 90 viable follicles were randomly assigned to six experimental groups. In three of these groups, follicles were cultured individually (1 follicle/well), whereas in the remaining three groups, grouped-follicles were cultured (4 follicles/well). Both individual (I) and group-cultured follicles (G) were either seeded directly onto the growth surface (0%-I, n = 6; 0%-G, n = 24), encapsulated in 0.5% sodium alginate (0.5%-I, n = 6; 0.5%-G, n = 24), or encapsulated in 1% sodium alginate (1%-I, n = 6; 1%-G, n = 24).

Each well of a 24-well plate (Nunclon Delta, ref. 142485) contained 400 μL of complete culture medium supplemented with 50 ng/mL FSH (porcine FSH, Prospec, ref. HOR-285, Ness-Ziona, Israel), 100 ng/mL EGF (Sigma-Aldrich, ref. E9644), and 100 ng/mL IGF-I (Sigma-Aldrich, ref. I3769). After nine days of culture, 50 ng/mL LH was added (porcine LH, Prospec, ref. HOR-310).

Follicles assigned to the non-encapsulated groups were directly transferred (one or four per well, according to the experimental design) into plates containing complete medium. For alginate-encapsulated follicles, each one was embedded in an alginate droplet as previously described [43], with slight modifications. Briefly, sodium alginate (65% guluronic acid; Sigma-Aldrich, ref. 71238) was dissolved in PBS at a final concentration of 0.5% or 1% (*w*/*v*). The cross-linking solution was prepared with 50 mM CaCl_2_ and 140 mM NaCl [29]. Both solutions were filtered through sterile 0.22 μm filters (MF-Millipore, Sigma-Aldrich, ref. SLGSR33SS), autoclaved, and subsequently stored at 4 °C. Three to four 40 μL drops of sodium alginate solution (0.5% or 1%) were placed on a Petri dish, and each follicle was transferred sequentially using <5 μL of medium. The last droplet containing the follicle was aspirated with 5 μL alginate and slowly released into 400 μL of cross-linking solution per well, then incubated at 37 °C and 5% CO_2_ for 5 min to form beads. Each encapsulated follicle was then transferred to a 24-well plate with 400 μL of complete medium, either 4 follicles/well (group culture) or 1 follicle/well (individual culture).

Follicles were cultured at 38.5 °C, with 5% CO_2_, and 99% humidity for 14 days. Culture medium was replaced every 48 h with fresh medium of identical composition. Collected media were stored at −20 °C for subsequent hormone analysis.

### 2.3. Follicular Morphometric Analysis

Follicle survival and growth were assessed every 24–48 h using an inverted microscope equipped with transmitted light and phase-contrast objectives (Ti-Eclipse, Nikon, Tokyo, Japan). Images were acquired and analyzed using an image analysis program (NIS-Elements, Nikon). Follicle diameter (μm) was determined by averaging two perpendicular measurements across the outer cell layer. Follicles were classified as degenerated when a measurable decrease in diameter occurred relative to earlier culture days, when granulosa cells displayed signs of fragmentation or detachment, or when the follicle no longer maintained its structural integrity, evidenced by oocyte detachment or loss of enclosure [39,44,45].

### 2.4. Histological Analysis

Follicles from three wells per group were collected after 7 and 14 days of culture by enzymatic degradation of the alginate beads using 10 IU/mL alginate lyase (Sigma-Aldrich, ref. A-1603) for 30 min at 37 °C under 5% CO_2_. Then, the digestion medium was replaced with PBS at 37 °C.

Fixation, dehydration, and double inclusion were performed according to a previous patented procedure (Patent P201300524/PCT/ES2014/000089). Briefly, follicles were washed twice for 5 min in Dulbecco’s PBS (DPBS, Sigma-Aldrich, ref. D8537) at 37 °C and fixed in 4% paraformaldehyde (pH 7.4) for 15 min at 4 °C. After washing twice with PBS, samples were dehydrated in ascending concentrations of ethanol (30%, 50%, and 70%; Merck Millipore, ref. 1009832500, Burlington, MA, USA) for 10 min each at 2–8 °C. These processes were conducted under a stereomicroscope (Nikon SMZ 800, Nikon) with the aid of micropipettes (Steripette, Minitube Ibérica, ref. 19025/0050, Tarragona, Spain).

Follicles were aggregated according to each group in 1.5% aqueous agar (Oxoid Ltd., ref. LP001; Hampshire, UK). The agar was dispensed into polypropylene moulds (5 mm diameter × 3 mm depth) to form a basal layer. Once solidified, follicles were transferred onto the agar layer and overlaid with additional molten agar to fully embed the clusters. The moulds were incubated at 2–8 °C for 3–4 h to ensure complete solidification. Agar blocks were retrieved using 26-gauge needles, placed in labeled histology cassettes, and stored in 70% ethanol until paraffin embedding. Paraffin-embedded samples were serially sectioned at 3 μm thickness, dewaxed with xylene, and rehydrated through decreasing concentrations of ethanol in aqueous solutions. Sections were then stained with hematoxylin and eosin (H&E), cleared, and mounted for histological evaluation. Images were captured using an optical microscope (Olympus BX50, Olympus, Tokyo, Japan) equipped with a camera (Olympus DP27, Olympus) and processed using Viewfinder Lite and Studio Lite, version 7.4.1. (Better Light, Inc., San Carlos, CA, USA) software.

### 2.5. Steroid Hormone Analyses

Estradiol (E2), testosterone (T), and progesterone (P4) concentrations were measured in culture media collected every 48 h using commercially available enzyme immunoassay (EIA) kits (Demeditec Diagnostics, GmbH, Kiel, Germany), previously validated in our laboratory, following the manufacturer’s protocols.

E2 was measured using the Estradiol ELISA kit (ref. DE4399; sensitivity = 1.39 pg/mL), T using the Testosterone ELISA kit (ref. DE1559; sensitivity = 0.083 ng/mL), and P4 using the Progesterone ELISA kit (ref. DE1561; sensitivity = 0.045 ng/mL). Medium from wells without follicles served as negative controls. Optical density was measured at 450 nm using a microplate reader (Stat Fax 3200, Awareness Technology Inc., Palm City, FL, USA).

### 2.6. Statistical Analyses

Data were analyzed using one-way analysis of variance (ANOVA) to evaluate the effects of alginate concentration and culture type (group vs. individual) on follicular diameter at each time point, as well as to assess the influence of culture time within each treatment group (0%-G, 0%-I, 0.5%-G, 0.5%-I, 1%-G, 1%-I). Post hoc comparisons were conducted using Bonferroni’s multiple comparison test. Student’s *t*-test was used to compare the percentages of intact follicles among groups within each time point.

One-way ANOVA was also applied to assess the effects of alginate concentration, either in group-cultured or individually cultured follicles, on steroid hormone levels at each time point, and to compare hormone concentrations within each group across the culture period. Post hoc comparisons were conducted using Duncan’s multiple range test. The same analysis approach was used, after normalization of hormone concentrations to the number of follicles per well, to compare hormone release per follicle among different groups and within each group over time.

Differences were considered statistically significant at *p* < 0.05, *p* < 0.01, or *p* < 0.001. All statistical analyses were performed using SAS software (version 9.4; SAS Institute Inc., Cary, NC, USA) with support from the Informatics Services, Complutense University of Madrid, and GraphPad Prism 4 (GraphPad Software Inc., San Diego, CA, USA).

## 3. Results

The following results describe the effects of alginate encapsulation and culture conditions on the morphological, morphometric, histological, and steroidogenic characteristics of porcine preantral follicles cultured in vitro.

### 3.1. The Culture Medium Markedly Influenced the 3D Organization of In Vitro-Developed Porcine Preantral Follicles

**Figure 1:**
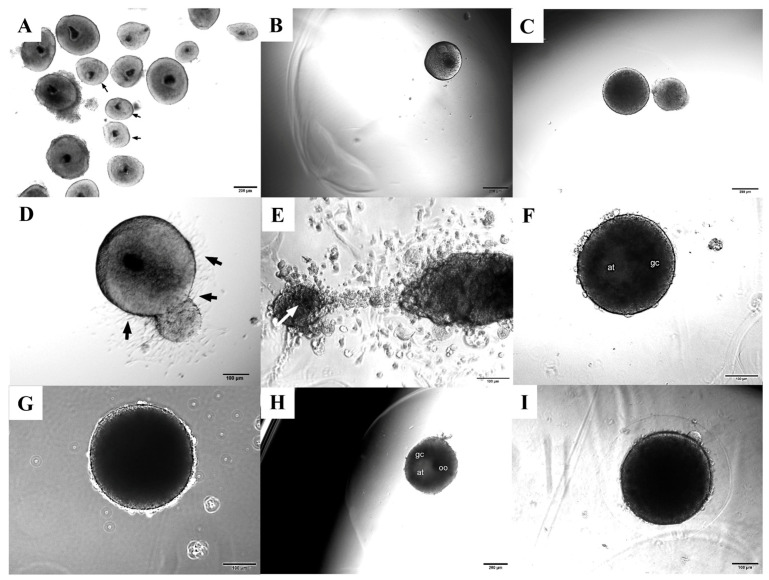
Microphotographs showing different morphological features of porcine preantral follicles during in vitro culture with or without alginate. Representative porcine eight-to-ten-layered secondary follicles (black arrows) from a subset of isolated follicles on day 1, showing the oocyte and surrounding granulosa cells; remaining follicles were excluded from the study due to inappropriate size or morphology ((**A**) 4×/0.10 magnification; Plan Achromat); representative isolated follicle on day 1 encapsulated within alginate hydrogel ((**B**) 4×/0.10 magnification; Plan Achromat); heterogeneous follicular growth within the same well ((**C**) 4×/0.10 magnification; Plan Achromat); from day 5 onward, granulosa cells began to migrate away from the oocyte (black arrows) in follicles directly seeded onto the culture surface ((**D**) 4×/0.10 magnification; Plan Achromat); extruded oocyte (white arrow) and cumulus cells on day 9 ((**E**) 10×/0.22 magnification, phase contrast); preantral follicle cultured in 0.5% alginate showing antrum formation on day 7 ((**F**) 10×/0.22 magnification, phase contrast); follicle cultured in 0.5% alginate (0.5%-G) on day 12 maintaining its spherical structure and exhibiting greater growth compared with the other groups ((**G**) 10×/0.22 magnification, phase contrast); preantral follicle cultured in 1% alginate showing the oocyte and antrum formation on day 7 ((**H**) 4×/0.10 magnification; Plan Achromat); follicle cultured in 1% alginate (1%-G) on day 12 maintaining its spherical morphology, although with smaller size compared with the 0.5% group ((**I**) 10×/0.22 magnification, phase contrast). oc = oocyte; gc = granulosa cells; at = antrum.

Although intact preantral follicles measuring 240–260 μm in diameter were initially selected for culture (Figure 1A,B), heterogeneous growth was observed in some wells as incubation progressed, with follicles of clearly different diameters developing within the same well (Figure 1C).

Follicles directly seeded onto the culture surface (groups 0%-G and 0%-I; Figure 1C–E) maintained their structural integrity during the initial days of culture. However, from day 5 onward, granulosa cells began to migrate away from the oocyte (Figure 1D) and frequently spread across the culture plate surface. By the later culture stages, oocyte extrusion from the follicle was occasionally observed (Figure 1E). In contrast, follicles cultured with 0.5% alginate hydrogels (groups 0.5%-G and 0.5%-I) generally preserved their 3D architecture and exhibited consistent growth throughout the culture period (Figure 1F,G). Most follicles displayed a clearly visible oocyte and zona pellucida during the first days of culture, followed by the onset of antrum formation approximately from day 5 onward (Figure 1F). Follicles embedded in 1% alginate (groups 1%-G and 1%-I) showed the follicular antrum as culture progressed (Figure 1H), also preserving their overall morphology throughout the culture period (Figure 1I).

**Figure 2:**
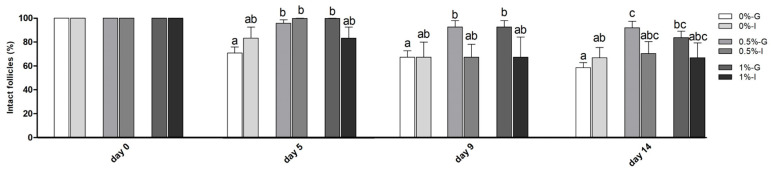
Percentages (mean ± SE) of morphologically intact follicles over 14 days of culture embedded in different alginate concentrations (0%, 0.5%, and 1%) for in vitro grouped (G) or individual (I) culture. Different letters (a, b, c) denote significant differences (*p* < 0.05) among groups at each time point.

The percentage of morphologically intact follicles throughout culture is shown in Figure 2. During days 5 and 9, this percentage was larger (*p* < 0.05) in the 0.5%-G and 1%-G groups compared with the 0%-G group. The highest values on day 14 were recorded in the 0.5%-G group (91.67 ± 5.27%), which were greater than those observed in the 0%-G (58.3 ± 4.21%, *p* < 0.001) and 0%-I (66.67 ± 12.36%, *p* < 0.05) groups. Likewise, follicles from the 1%-G group (83.33 ± 5.30%) showed larger values (*p* < 0.01) than follicles from the 0%-G group on day 14.

### 3.2. The In Vitro Development of Porcine Preantral Follicles Was Influenced by Alginate Encapsulation During the 14-Day Culture Period

**Figure 3:**
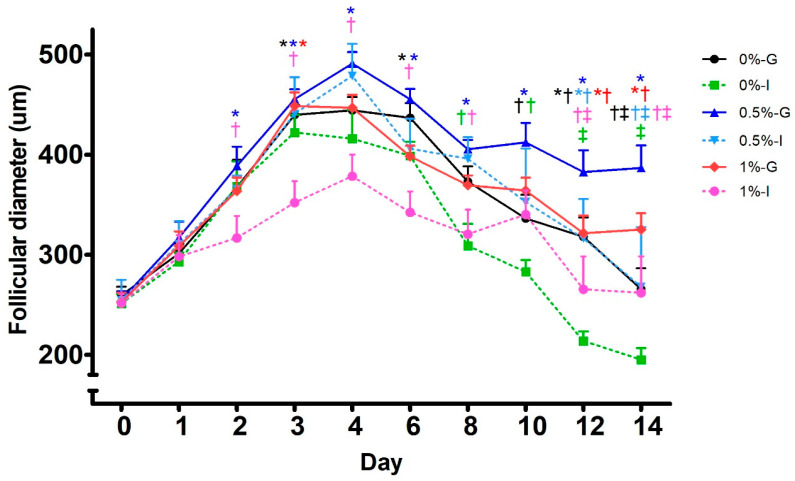
Line-graph representation of the time-course follicular development of porcine preantral follicles cultured for 14 days at different alginate concentrations (0%, 0.5%, and 1%) under grouped (G) or individual (I) culture conditions. Different symbols (*, †, ‡) denote significant differences (*p* < 0.05) among treatments at each time point; colors correspond to those described in the legend.

Follicular diameters increased progressively in all groups up to day 4, with growth being more pronounced in group-cultured follicles than in individually cultured follicles (Figure 3). From days 2 to 4, follicles in the 1%-I group exhibited smaller diameters (*p* < 0.05) than those in the 0.5%-G group. On day 3, follicular size in the 1%-I group was also reduced relative to the 0%-G (*p* < 0.05) and 1%-G (*p* < 0.01) groups. From days 4 to 8, follicular growth plateaued, with larger diameters in the 0.5%-G group compared with 1%-I (*p* < 0.01) and 0%-I (*p* < 0.001) on day 8. Follicles from 0%-G were also larger than those from 1%-I (*p* < 0.01) on day 6 (Figure 3). From day 10 onward, coinciding with LH addition, follicular diameters in the 0.5%-G group, and to a lesser extent in 1%-G, remained stable and were greater (*p* < 0.05) than those in the other groups at the end of culture. Follicles in 0.5%-G were larger than those in 0%-I from days 10 to 14 (*p* < 0.001), in 0%-G on days 10 and 14 (*p* < 0.05), in 0.5%-I on day 14 (*p* < 0.01), and in 1%-I on days 12 and 14 (*p* < 0.05). Similarly, 1%-G follicles were larger than those of 0%-I from days 12 to 14 (*p* < 0.01). Follicles from 0%-I reached also smaller diameters (*p* < 0.05) than 0%-G and 0.5%-I on day 12 (Figure 3).

### 3.3. Histological Analysis Revealed Significant Differences Between Follicles Cultured in a 2D System and Those Maintained in an Alginate-Based 3D Matrix

**Figure 4:**
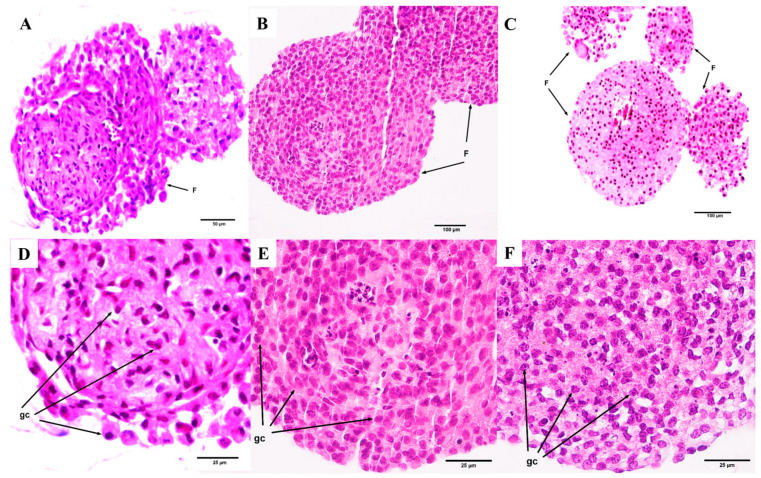
Microphotographs showing representative histological features of porcine preantral follicles on day 7 under different culture conditions (H&E staining). Follicles directly seeded onto the 2D culture surface exhibited a marked loss of spherical architecture, with granulosa cells spreading across the substrate and showing signs of cellular degeneration, including pyknosis and abnormal intercellular spacing ((**A**) 20×/0.70 magnification; (**D**) 40×/0.75 magnification, higher-magnification view of (**A**)). In contrast, follicles cultured within the alginate-based 3D matrix in 0.5% alginate ((**B**) 10×/0.30 magnification; (**E**) 40×/0.75 magnification, higher-magnification view of (**B**)) and 1% alginate ((**C**) 10×/0.30 magnification; (**F**) 40×/0.75 magnification) preserved a well-defined spherical morphology, with granulosa cells remaining compact. F = follicle; gc = granulosa cells.

Follicles directly seeded onto the culture surface (Figure 4A,D) exhibited a clear loss of their spherical architecture, frequently adopting flattened or irregular shapes. Granulosa cells tended to disperse and spread across the culture surface, resulting in reduced cellular cohesion. In some cases, evidence of cellular degeneration was observed, including pyknosis and abnormal intercellular spacing.

In contrast, follicles cultured within the alginate 3D matrix (Figure 4B,C,E,F) preserved a well-defined spherical morphology, closely resembling in vivo structures. Granulosa cells remained compact, maintaining the integrity of cell–cell interactions, with a notably lower incidence of degenerative features.

### 3.4. Encapsulating Porcine Preantral Follicles in Alginate Hydrogels Influenced Their Steroidogenic Activity Throughout the Culture Period

**Figure 5:**
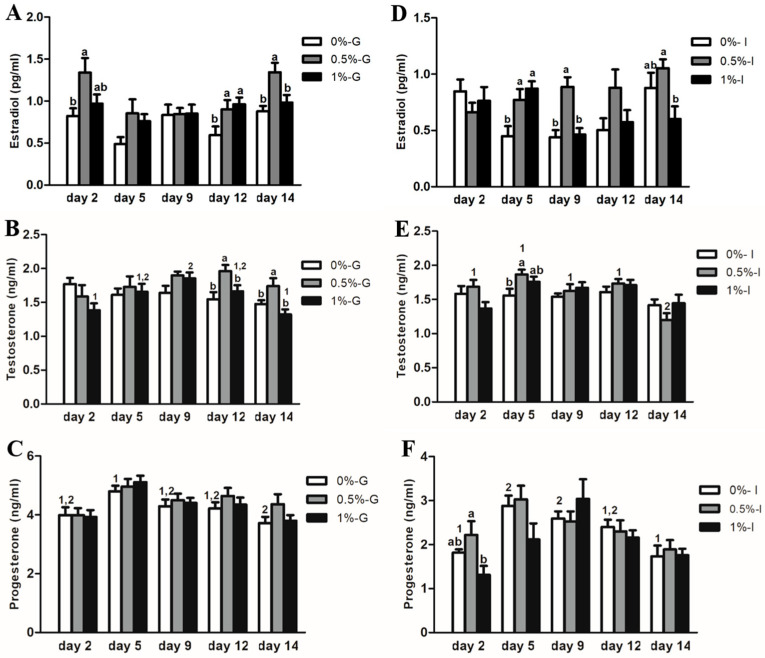
Bar graphs depicting estradiol (pg/mL), testosterone (ng/mL), and progesterone (ng/mL) concentrations on days 2, 5, 9, 12, and 14 of culture in 0%-G, 0.5%-G, 1%-G, 0%-I, 0.5%-I, and 1%-I groups. (**A**–**C**) represent estradiol, testosterone, and progesterone concentrations, respectively, in group-cultured follicles (G); (**D**–**F**) represent estradiol, testosterone, and progesterone concentrations, respectively, in individually cultured follicles (I). Different letters (a, b) denote significant differences (*p* < 0.05) among groups at each time point. Different numbers (1, 2) denote significant differences (*p* < 0.05) within each group over the culture period.

In group-cultured follicles, E2 concentrations were higher (*p* < 0.05) in the 0.5%-G group compared with the 0%-G group on days 2, 12, and 14 (from 1.344 ± 0.11 pg/mL to 0.877 ± 0.07 pg/mL on day 14, respectively), and larger than in the 1%-G group on day 14 (0.981 ± 0.09 pg/mL) (Figure 5A). E2 concentrations in the 1%-G group were also greater than those in the 0%-G group on day 12 (*p* < 0.05). T concentrations were elevated (*p* < 0.05) in the 0.5%-G group compared with 0%-G and 1%-G on days 12 and 14 (from 1.740 ± 0.12 µg/mL in 0.5%-G to 1.475 ± 0.06 µg/mL in 0%-G and 1.321 ± 0.08 µg/mL in 1%-G on day 14). Within the 1%-G group, T levels increased (*p* < 0.05) on day 9 compared with days 2 and 14 (Figure 5B). P4 concentrations were higher (*p* < 0.05) on day 5 over values quantified on day 14 in the 0%-G group (Figure 5C).

In individually cultured follicles, E2 concentrations were greater (*p* < 0.05) in the 0.5%-I group than in the 0%-I group on days 5 and 9 (from 0.887 ± 0.08 pg/mL to 0.439 ± 0.06 pg/mL on day 9, respectively), and with the 1%-I group on days 9 and 14 (from 1.054 ± 0.08 pg/mL to 0.602 ± 0.11 pg/mL on day 14, respectively) (Figure 5D). T concentrations were elevated (*p* < 0.05) on day 5 in the 0.5%-I group compared with the 0%-I (from 1.865 ± 0.07 µg/mL to 1.560 ± 0.10 µg/mL, respectively). Within the 0.5%-I group, T concentrations were lower (*p* < 0.05) on day 14 than at earlier time points (Figure 5E). P4 concentrations were larger in the 0.5%-I group than in the 1%-I group on day 2 (from 2.219 ± 0.31 µg/mL to 1.311 ± 0.20 µg/mL, respectively), whereas within the 0%-I group, P4 levels were increased (*p* < 0.05) on days 5 and 9 compared with days 2 and 14 (Figure 5F).

Per-follicle-normalized steroid concentrations in conditioned medium at each time point increased in individually cultured follicles (0%-I, 0.5%-I, 1%-I) when compared with group-cultured follicles (0%-G, 0.5%-G, 1%-G) (Table 1). Estimated E2 concentrations secreted per follicle were larger (*p* < 0.05) in individually cultured follicles than in group-cultured follicles, with the highest observed in 0.5%-I compared with 0%-I on days 5 and 9, and with 1%-I on days 9 and 14. T concentrations secreted per follicle were also greater (*p* < 0.001) in individually cultured follicles throughout the culture period. Similarly, P4 concentrations secreted per follicle were greater in individually cultured follicles, particularly on days 5, 9, and 12, when values were higher (*p* < 0.05) than those in the 0.5%-G and 1%-G groups.

## 4. Discussion

The present study demonstrates for the first time that encapsulation of porcine preantral follicles in alginate hydrogel for 3D culture, as well as culturing grouped or individualized follicles, significantly influences follicular morphology, time-course development, and steroid hormone production during a 14-day in vitro culture period.

Follicles seeded directly onto growth surfaces without alginate (0%) progressively lost their 3D architecture, with granulosa cell migration observed from day 5 onward and occasional oocyte extrusion. These findings align with previous studies showing that 2D culture causes follicular flattening and disrupts the cell–cell interactions essential for autocrine and paracrine signaling between the oocyte and somatic cells [46]. This communication depends on connexin-based gap junctions that allow the exchange of ions and small molecules [47,48], which are compromised in 2D systems, leading to impaired intercellular signalling and follicular development. Consequently, the alginate-free group showed the lowest proportion of morphologically intact follicles at the end of culture, ranging from 58.3 to 66.7%.

In contrast, 3D alginate encapsulation preserved follicular spherical architecture, resulting in larger percentages of morphologically intact follicles at the end of the culture. Previous studies have shown that alginate-based 3D systems maintain follicular structure and oocyte–somatic cell communication [25,27,49]. Notably, the 0.5%-G group exhibited the best structural integrity on day 14 (91.7 ± 5.27%), indicating an optimal balance between mechanical support and diffusional permeability. Although the optimal matrix concentration may vary among species, consistent with previous reports, 0.5% alginate would closely mimic the ovarian mechanical microenvironment, supporting granulosa cell proliferation, follicle expansion, antrum formation, and preserved cell–cell communication in preantral follicles [25,32,34].

Although follicle survival in the 1%-G group was slightly lower than in the 0.5%-G group, it remained high (83.3 ± 5.30%), aligning with reports showing minimal effects of alginate concentration on viability [50]. However, despite preserving follicular architecture, the stiffer 1% matrix appeared to constrain follicle growth.

Morphometric analysis showed that all groups exhibited an initial increase in follicular diameter up to day 4, corresponding to the transition from the gonadotropin-independent to the gonadotropin-responsive phase, predominantly regulated by intra-ovarian factors that can be stimulated by FSH [16]. The oocyte and the surrounding granulosa cells grow and differentiate in response to local factors, although the addition of FSH at this stage appeared to further stimulate follicular growth. During this period, most follicles maintained their structural integrity, suggesting preserved intercellular connections.

Follicular growth stabilized between days 4 and 8; however, following LH supplementation, follicles in the 0.5%-G group, and to a lesser extent the 1%-G group, continued to exhibit larger diameters. This period corresponds to the transition from the gonadotropin-responsive to the gonadotropin-dependent phase, during which follicles acquire LH responsiveness [16]. This suggests that follicles cultured in 0.5% alginate would have preserved both structural integrity and functional responsiveness to LH, consistent with a permissive 3D microenvironment that supports developmental capacity and responsiveness to gonadotropin signals.

In contrast, follicles cultured without alginate or within a stiffer matrix (1%) exhibited limited growth. As discussed above, in the absence of alginate, follicular architecture gets disrupted, leading to granulosa cell displacement and follicular flattening, which may result in measurements that do not accurately reflect the true 3D structure of the follicle [34]. Excessive matrix stiffness may restrict follicular expansion through compressive forces, reduced nutrient and hormone diffusion, and impaired paracrine signaling [24,51], ultimately inhibiting follicular and oocyte development and downregulating key follicular genes [29,49,50,52]. Notably, early folliculogenesis occurs within the relatively rigid ovarian cortex, whereas continued development involves migration toward the softer medullary region, which permits further follicular expansion [53].

Follicular development was more efficient in group-cultured follicles than in isolated follicles, supporting the concept that inter-follicular communication enhances maturation. In consistency with previous studies, co-culture of multiple follicles likely stimulates growth through paracrine signaling and the exchange of growth-promoting factors [12,39,54], preserving granulosa cell organization, gap-junction integrity, and meiotic competence [55].

Steroid hormone analyses further supported these findings, as group-cultured follicles encapsulated in 0.5% alginate produced significantly larger E2 and T concentrations than those in 0%-G and 1%-G groups, suggesting that optimal matrix stiffness enhances steroidogenic capacity. Softer matrices have been associated with improved steroid secretion across species [24,32,34], up-regulating the expression of genes involved in steroidogenesis [56], whereas increased stiffness disrupts granulosa and theca cell differentiation, limits diffusion, and impairs paracrine and autocrine signaling, ultimately reducing steroidogenic gene expression and gonadotropin responsiveness [23,24].

In the current study, follicles cultured in 0.5% alginate secreted greater E2 and T concentrations to conditioned medium, whereas P4 levels were comparable to those in 1% alginate, in agreement with previous research showing that permissive matrices favor estrogen production and efficient steroid conversion [50]. In contrast, stiffer matrices are associated with reduced E2, elevated P4 and androgens, and premature follicle luteinization, reflecting impaired intercellular communication and altered follicular function [24,32]. Beyond providing mechanical support, alginate serves as a regulatory microenvironment influencing paracrine and autocrine signaling. Stiffer matrices impair communication among granulosa, theca, and oocyte cells, disrupting steroidogenesis and androgen to E2 conversion, while softer matrices facilitate nutrient and hormone exchange, supporting 3D follicle growth [57]. Therefore, stiff matrices are associated with elevated androstenedione and P4, reduced E2, due to inefficient steroid conversion [24], and premature luteinization of follicular cells [45]. Optimal matrix rigidity varies with species and follicular stage, with primordial follicles often requiring relatively stiffer environments.

Conversely, in 2D culture, follicular flattening and granulosa cell migration disrupt oocyte–granulosa communication, impairing gap junction and paracrine/autocrine signaling, and reducing steroidogenesis, as evidenced by lower E2 and T concentrations [56,58].

At the end of the culture period, individually cultured follicles displayed smaller diameters and lower hormone output than group-cultured follicles. However, when normalized per follicle, steroid concentrations quantified in conditioned medium increased, particularly in the 0.5%-I group, suggesting enhanced steroidogenic efficiency on a per-follicle basis. Group culture promotes collective growth through paracrine factors [39] such as Kit ligand, activin, BMP-2/5, EGF, FGF-8, and growth differentiation factor-9 (GDF-9), which stimulate proliferation of granulosa and theca cells and support early follicle growth [59,60,61,62]. High local concentrations of these growth-promoting signals may sustain granulosa cell mitotic activity, increasing follicle size while potentially delaying the transition to a fully differentiated, steroidogenically mature state. Since the acquisition of steroidogenic competence depends on granulosa cell differentiation, prolonged proliferation can result in lower hormone output per cell despite larger follicle diameters.

As culture progressed, heterogeneous growth was occasionally observed within the same well, with follicles displaying markedly different diameters despite initially being of similar size. This resembles the physiological interactions among preantral follicles during early folliculogenesis in the ovarian cortex, where growing preantral follicles secrete anti-Müllerian hormone (AMH) to locally inhibit the activation and growth of neighboring follicles, promoting asymmetric development [63] to preserve the oocyte pool. In vitro group cultures may reflect these dynamics, generating cohort heterogeneity [64]. Future experiments will address these issues.

Overall, group culture enhances follicular viability and development by providing paracrine support and better mimicking the in vivo environment, making it suitable for long-term follicle culture. In contrast, individual culture remains appropriate for short-term experiments, as it allows follicle growth to be easily monitored through daily measurements [11,40].

The present study has certain limitations. While it focused on follicular morphology, growth, and steroidogenesis, oocyte developmental competence, a critical endpoint for translational relevance, was not assessed. Additionally, future investigations should explore co-culture systems incorporating somatic cells or the use of composite biomaterials. Notably, hybrid hydrogels combining alginate with fibrin or hyaluronate further enhance steroidogenesis in goat and sheep follicle cultures [58], highlighting their potential to optimize the follicular microenvironment. In addition, testing intermediate alginate concentrations, such as 0.25% or 0.75%, and performing immunohistochemical analyses would provide further insight into follicular development and function. Moreover, future studies should incorporate molecular analyses of follicle-related functional genes to further support and validate follicular functionality under the optimized culture conditions.

## 5. Conclusions

This study demonstrates for the first time that encapsulation of porcine preantral follicles in 0.5% alginate hydrogels for 3D culture provides an optimal balance between mechanical support and molecular diffusion, preserving follicular integrity, enhancing development, and maintaining gonadotropin responsiveness. In contrast, both the 2D culture system and the apparently excessive matrix stiffness after encapsulation of these follicles in 1% alginate hydrogel disrupted follicular architecture, limited growth, and impaired steroidogenesis. Group-cultured follicles enhanced viability and collective hormone secretion via paracrine interactions, whereas individually cultured follicles increased per-follicle steroidogenic efficiency. Together, these findings underscore the importance of fine-tuning both hydrogel composition and culture system to accurately replicate the ovarian microenvironment and optimize porcine in vitro follicle development for applications in reproductive biotechnology and fertility preservation.

## Figures and Tables

**Table 1:** Steroid hormone concentrations normalized to the number of follicles cultured per well at each time point in individually cultured porcine follicles (0%-I, 0.5%-I, 1%-I) and group-cultured follicles (0%-G, 0.5%-G, 1%-G) throughout the in vitro culture period. Different letters (a, b, c) indicate statistically significant differences among groups at each time point (*p* < 0.05).

**Estradiol** **(pg/mL)**	**Day 2**	**Day 5**	**Day 9**	**Day 12**	**Day 14**
0%-G	0.29 ± 0.08 a	0.20 ± 0.08 a	0.31 ± 0.11 ac	0.26 ± 0.11 a	0.38 ± 0.07 a
0%-I	0.85 ± 0.26 b	0.45 ± 0.22 a	0.44 ± 0.16 ac	0.51 ± 0.25 ab	0.88 ± 0.33 bc
0.5%-G	0.35 ± 0.11 a	0.23 ± 0.11 a	0.23 ± 0.05 ac	0.24 ± 0.07 a	0.36 ± 0.07 a
0.5%-I	0.66 ± 0.21 b	0.77 ± 0.23 b	0.89 ± 0.21 b	0.57 ± 0.26 ab	0.60 ± 0.28 b
1%-G	0.24 ± 0.07 a	0.19 ± 0.05 a	0.21 ± 0.07 a	0.24 ± 0.05 a	0.30 ± 0.07 a
1%-I	0.76 ± 0.30 b	0.87 ± 0.16 b	0.46 ± 0.14 c	0.88 ± 0.40 b	1.05 ± 0.19 c
**Testosterone** **(µg/mL)**	**Day 2**	**Day 5**	**Day 9**	**Day 12**	**Day 14**
0%-G	0.63 ± 0.08 a	0.58 ± 0.08 a	0.61 ± 0.09 a	0.67 ± 0.11 a	0.64 ± 0.06 a
0%-I	1.6 ± 0.28 b	1.6 ± 0.24 b	1.5 ± 0.12 b	1.60 ± 0.19 b	1.40 ± 0.21 b
0.5%-G	0.42 ± 0.11 a	0.45 ± 0.10 a	0.51 ± 0.04 a	0.53 ± 0.06 ac	0.47 ± 0.08 a
0.5%-I	1.69 ± 0.24 b	1.87 ± 0.17 c	1.63 ± 0.24 b	1.73 ± 0.16 b	1.19 ± 0.25 b
1%-G	0.35 ± 0.06 a	0.41 ± 0.07 a	0.46 ± 0.05 a	0.42 ± 0.05 c	0.40 ± 0.06 a
1%-I	1.37 ± 0.23 b	1.76 ± 0.19 bc	1.67 ± 0.20 b	1.71 ± 0.18 b	1.44 ± 0.31 b
**Progesterone (µg/mL)**	**Day 2**	**Day 5**	**Day 9**	**Day 12**	**Day 14**
0%-G	1.43 ± 0.23 ac	1.71 ± 0.17 a	1.59 ± 0.21 a	1.83 ± 0.23 ac	1.62 ± 0.23 ab
0%-I	1.81 ± 0.19 ab	2.88 ± 0.57 b	2.60 ± 0.39 b	2.41 ± 0.41 a	1.73 ± 0.61 ab
0.5%-G	1.05 ± 0.15 c	1.31 ± 0.17 a	1.22 ± 0.15 a	1.25 ± 0.18 bc	1.18 ± 0.23 a
0.5%-I	2.22 ± 0.77 b	3.03 ± 0.76 b	2.53 ± 0.55 b	2.30 ± 0.61 a	1.89 ± 0.52 b
1%-G	0.9 8± 0.14 c	1.28 ± 0.14 a	1.10 ± 0.11 a	1.08 ± 0.15 b	1.15 ± 0.14 a
1%-I	1.31 ± 0.50 ac	2.12 ± 0.89 ab	3.04 ± 1.09 b	2.16 ± 0.41 a	1.76 ± 0.36 ab

## Data Availability

Data are available from the corresponding author upon reasonable request.
